# Lipid-based nano-formulation platform for eplerenone oral delivery as a potential treatment of chronic central serous chorioretinopathy: *in-vitro* optimization and *ex-vivo* assessment

**DOI:** 10.1080/10717544.2021.1902023

**Published:** 2021-03-31

**Authors:** Eman Abdelhakeem, Mohamed El-Nabarawi, Rehab Shamma

**Affiliations:** Department of Pharmaceutics and Industrial Pharmacy, Faculty of Pharmacy, Cairo University, Cairo, Egypt

**Keywords:** Eplerenone, nanostructured lipid carriers, oral delivery, vesicular uptake

## Abstract

**Purpose:**

Eplerenone (EPL) is a selective mineralocorticoid receptor antagonist used for treatment of chronic central serous chorioretinopathy which characterized by accumulation of subretinal fluid causing a localized area of retinal detachment. unfortunately, EPL suffers from poor oral bioavailability due to poor aqueous solubility in addition to high hepatic first pass metabolism.

**Method:**

Aiming to improve its oral bioavailability, EPL-loaded nanostructured lipid carriers (NLCs) were prepared by the emulsification solvent evaporation method and *in-vitro* evaluated for particle size (PS), polydispersity index (PDI), zeta potential (ZP), and entrapment efficiency (EE%). A D-optimal design was used for study the effect of liquid lipid to solid lipid ratio, surfactant type and percentage on PS, PDI, EE%, and for data optimization. The optimized EPL-loaded NLCs system was further evaluated using *in-vitro* drug release and *ex-vivo* permeation studies through rabbit intestine in comparison to EPL aqueous suspension. The physicochemical properties of the drug in the optimized system were further examined using FT-IR and X-ray diffraction studies.

**Results:**

The resultant NLCs showed small PS (100.85–346.60 nm), homogenous distribution (0.173–0.624), negatively charged particles (ZP −20.20 to −36.75 mV), in addition to EE% (34.31–70.64%). The optimized EPL-loaded NLCs system with a desirability value of 0.905 was suggested through the Design expert^®^ software, containing liquid to solid lipid ratio (2:1) in presence of 0.43%w/v Pluronic^®^ F127 as a surfactant. The optimized EPL-loaded NLCs system showed a PS of 134 nm and PDI of 0.31, in addition to high EE% (76 ± 6.56%w/w), and ZP (-32.37 mV). The *ex-vivo* permeation study showed two-fold higher drug permeation through rabbit intestine compared to that from the aqueous drug suspension after 24 h, confirming the ability of optimized EPL-loaded NLCs system as successful oral targeting delivery carrier.

**Conclusion:**

Our results pave the way for a new oral nanotherapeutic approach toward CSCR treatment. *In-vivo* study is currently under investigation.

## 1. Introduction

Chronic central serous chorioretinopathy (CSCR), a vision hostile disease, is a common cause of visual impairment in the working-age population and has been estimated as the fourth most frequently encountered retinopathy after age-related macular degeneration, diabetic retinopathy, and retinal vein occlusion (Gemenetzi et al., 2010; Salehi et al., 2015). CSCR is characterized by accumulation of the subretinal fluid causing a localized area of retinal detachment (Bouzas et al., 2002; Bousquet et al., 2013). Unfortunately, the causative factor behind CSCR is not completely understood (idiopathic).

The use of mineralocorticoid receptor antagonist in CSCR treatment refers to the existence of an independent renin–angiotensin aldosterone system at the ocular level. This system has greater expression in retinal and uveal tissue and acts on the ocular vasculature, aqueous humor, and intraocular pressure control (Savaskan et al., 2004; Nakashima et al., 2006).

Eplerenone (EPL) is a selective mineralocorticoid receptor antagonist. EPL is analogues to the commonly used diuretic spironolactone, with an increased mineralocorticoid receptor selectivity and higher affinity (Cook et al., 2003; Yang and Eliott, 2017). It has the ability to act at neuroretinal cell types (Zhao et al., 2010) modifying the above-mentioned physiopathological processes (Yang and Eliott, 2017; Campos Polo et al., 2018; Chatziralli et al., 2018).

Multiple studies have investigated the success and safety of oral EPL in the treatment of patients with chronic CSCR (Breukink et al., 2014; Salz et al., 2015; Chin et al., 2015; Pichi et al., 2015; Daruich et al., 2016; Pichi et al., 2017), where a rapid subretinal fluid resolution and improvement in the visual acuity have been observed (Zhao et al., 2012; Haghighi et al., 2018). Unfortunately, EPL is class II drug according to biopharmaceutical classification system with limited aqueous solubility (less than 1 mg/mL) (Khames, 2019), having a maximum aqueous solubility of 0.00908 mg/ml only (Kendre & Chaudhari, 2017), in addition to being extensively metabolized into the liver to inactive metabolites (Almeida et al., 2011; Salehi et al., 2015). This leads to poor oral bioavailability and consequently low therapeutic response (Özdemir et al., 2018b). Previous studies succeeded to improve its oral bioavailability and biodistribution through the formulation of EPL nano-emulsion and EPL solid dispersion (Almeida et al. 2011; Kendre & Chaudhari, 2017; Haghighi et al., 2018; Özdemir et al., 2018a). EPL oral bioadhesive pellets succeeded to improve EPL solubility and hence the bioavailability (Kendre & Chaudhari, 2017).

Nanostructured lipid carriers (NLCs) are modified solid lipid nanoparticles, compromising solid lipid and liquid lipid (oil) matrix, such like a regime enriched fat. After oral administration, these lipids have the ability to impel the bile secretion in the small intestine then the NLCs loaded with the drug become coupled with bile salts to form mixed micelles (Khan et al., 2015), allowing the intact NLCs to be transferred directly to the portal circulation through paracellular route or become gripped by microfold cells avoiding the first pass metabolism leading to a higher oral bioavailability (Khan et al., 2015; Ghasemiyeh & Mohammadi-Samani, 2018). In addition, NLCs with certain surfactants and lipids have been reported to efficiently avoid the efflux by P-glycoprotein and hence improvement in oral drug absorption (Bayón-Cordero et al., 2019). In addition, this unique composition allows to initiate a less organized structure diminishing the delocalization of the encapsulated drug and improving the loading ability (Radtke et al., 2005). Hence, NLCs are superior over other traditional carriers regarding enhancing the solubility of hydrophobic drugs, imparting controlled and sustained drug release characteristics and enhancing drug permeability. Moreover, NLCs could maintain the stability of drug during storage, by protecting it from chemical or enzymatic degradation (Radtke et al., 2005; Fang et al., 2012; Khan et al., 2015).

In our study, EPL-loaded NLCs were formulated by emulsification solvent evaporation technique (Shahgaldian et al., 2003), using glyceryl monostearate (GMS) as solid lipid and Miglyol^®^812 as liquid lipid. A d-optimal statistical study design was employed to study the effects of different formulation, and process variables and their interaction with limited number of experimental runs. The particle size, polydispersity index, zeta potential, and drug entrapment within the prepared NLCs were evaluated. Furthermore, the physicochemical properties of the drug within the optimized NLCs system were studied using infrared spectroscopy and X-ray diffraction. The morphology of the prepared NLCs was evaluated using TEM. Finally, an *ex-vivo* permeation study through rabbit intestine was performed to prove the capability of the optimized system to cross the intestinal mucosa following oral delivery of EPL. To our knowledge, this is the first study to fabricate oral EPL-loaded NLCs as a potential treatment for CSCR.

## 2. Materials and methods

### 2.1. Materials

Eplerenone was obtained from Shenzhen Oriental Pharmaceutical Co., Ltd., Guangdong, China. Glyceryl monostearate (Imwitor^®^900K) was purchased from Changwei Pharmaceutical Excipients Technology Co., Ltd. (Shanghai, China). Cremophor^®^ RH40 (Polyoxyl 40 Hydrogenated Castor Oil USP/NF), Pluronic^®^ F127 and Miglyol^®^81N2 were purchased from BASF chemical company (Ludwigshafen, Germany). Solutol^®^HS15, Disodium hydrogen phosphate, Sodium chloride and Potassium dihydrogen phosphate were purchased from Sigma Aldrich VR (St. Louis, MO). Ethanol and acetone were obtained from El-Nasr Pharmaceutical Chemicals, Cairo, Egypt. All other chemicals were of analytical grade and were used as received.

### 2.2. Preparation of EPL-loaded NLCs

EPL-loaded NLCs were prepared using the emulsification solvent evaporation technique using glyceryl monostearate (GMS) as solid lipid and Miglyol^®^812N as liquid lipid. In brief, 25 mg of EPL were dispersed in the determined amount of Miglyol^®^812N, then added to determined amount of molten GMS kept at 80°C using thermostatically controlled magnetic stirrer (WiseStir, Wisd Lab. Instruments, Tulsa, OK). Exactly, 10 mL mixture of ethanol and acetone (1:1 v/v) were added to the molten lipids maintained at 80 °C and stirred until complete dissolution in the organic phase. The organic phase was then added to 20 mL of an aqueous solution containing the chosen surface-active agent(s) to form a primary o/w emulsion under stirring at 1000 rpm for 1 min using magnetic stirrer. The obtained emulsion was subsequently subjected to 3 min of sonication using probe sonicator at room temperature (Probe Sonicator Ultrasonic Processor model VCX 750, Newtown, CT) adjusted at 40 W. Following this, the formed emulsion was stirred using magnetic stirrer at 500 rpm for 2 h at room temperature to allow evaporation of the organic solvent and formation of NLCs (Aburahma et al., 2014). Each formula was prepared three times, and the results are presented as mean ± SD (*n* = 3).

### 2.3. Evaluation of the prepared EPL-loaded NLCs

#### 2.3.1. Determination of particle size, polydispersity index, and zeta potential

The mean particle size (PS), polydispersity index (PDI), and zeta potential (ZP) were determined using dynamic light scattering (DLS, Malvern Zetasizer, Malvern, UK) at 25 °C (Imam et al., 2015). Before measurement, 0.1 mL of the dispersion was properly diluted with distilled water (10 mL) in a glass tube and shaken to have an appropriate scattering intensity. Data presented as mean values (*n* = 3± SD).

#### 2.3.2. Entrapment efficiency

The entrapment efficiency **(**EE% w/w) of EPL in the prepared EPL-loaded NLCs was determined indirectly by measuring the concentration of free drug in the aqueous phase of the NLCs dispersion. A definite volume (1 mL) of the prepared NLCs dispersion was centrifuged using cooling centrifuge (Sigma 3-30 KS, Sigma Laborzentrifugen GmbH, Osterode am Harz, Germany) at 22,000 rpm for 1 h at 4 °C. The supernatant was separated and properly diluted with ethanol, then the un-entrapped drug concentration was estimated spectrophotometrically at *λ*_max_ 241 nm. The EE% was calculated using the following equation:

$$\mathrm{The}\;EE\%=\frac{W_{\mathrm{initial}}-W_{\mathrm{free}}}{W_{\mathrm{initial}}}\times 100$$

where *W*_initial_ is the initial drug amount used in the preparation, and *W*_free_ is the un-entrapped drug amount.

### 2.4. Statistical design and optimization of EPL-loaded NLCs

The response surface methodology with polynomial equations is a beneficial numerical mean to investigate the effect of independent variables on the dependent variables (responses) based on a limited number of trails (Heurtault et al., 2003; Huang et al., 2004). Using d-optimal mixture design, maximum prediction power could be obtained in selected set of experimental runs, minimizing the discrepancy associated with estimates of coefficients in the model (Bodea & Leucuta, 1997). A d-optimal statistical design was employed to study the effect of different formulation variables on the prepared EPL-loaded NLCs. In this design, three liquid lipid to solid lipid ratios (1:1, 1:1.5, and 1:2 w/w), three surfactant types (Pluronic^®^ F127, Cremophor^®^ RH40 and Solutol^®^HS15) and three concentrations of surfactant (0.2, 0.4, and 0.6%w/v) were evaluated. Table 1 shows the different levels of the studied factors as well as the constraints applied in selection of the optimized EPL-loaded NLCs (minimize the PS, maximize the EE % and for the PDI to be less than 0.5). The composition of the prepared EPL-loaded NLCs is presented in Table 2 along with the measured responses. For this study, 19 runs were considered.

**Table 1.** Factors studied and respective levels investigated in the d-optimal design used for the preparation of EPL-loaded NLCs along with the responses and their required constraints.

Factors	Levels		
A: Liquid lipid to solid lipid ratio (w/w)	1:1	1.5:1	2:1
B: Conc. of surfactant (% w/v)	0.2	0.4	0.6
C: Type of surfactant	Cremophor^®^RH40	Solutol^®^HS15	Pluronic^®^ F127
Responses	Constraints		
Particle size (nm)	Minimize		
Polydispersity index	˂0.5		
Zeta potential (mV)	None		
Entrapment efficiency (%w/w)	Maximize		

**Table 2.** Composition of the prepared EPL-loaded NLCs and their own responses.

Formulation	A: Liquid lipid to solid lipid ratio (w/w)	B: Conc. of surfactant (% w/v)	C: Type of surfactant	Y1: PS (nm)	Y2: PDI	Y3: ZP (mV)	Y4: EE % (% w/w)
NLC 1	1:1	0.2	Cremophor^®^ RH40	215.06 ± 163.81	0.44 ± 0.22	−33.950 ± 0.212	52.23 ± 1.47
NLC 2	1.5:1	0.4	PL^®^-F127	207.13 ± 93.14	0.40 ± 0.01	−34.150 ± 0.212	70.48 ± 1.52
NLC 3	2:1	0.2	Solutol^®^HS15	187.30 ± 15.98	0.46 ± 0.08	−33.650 ± 0.212	34.46 ± 2.98
NLC 4	2:1	0.6	Solutol^®^HS15	200.40 ± 0.84	0.29 ± 0.01	−33.900 ± 1.980	46.20 ± 4.85
NLC 5	1:1	0.6	PL^®^-F127	346.60 ± 16.82	0.48 ± 0.15	−31.200 ± 1.273	62.44 ± 2.97
NLC 6	2:1	0.2	PL^®^-F127	104.46 ± 6.97	0.33 ± 0.01	−33.650 ± 1.202	49.25 ± 1.85
NLC 7	1:1	0.6	Cremophor^®^ RH40	258.01 ± 113.87	0.62 ± 0.03	−28.950 ± 0.071	43.95 ± 2.02
NLC 8	1:1	0.2	PL^®^-F127	195.05 ± 22.69	0.58 ± 0.08	−28.100 ± 4.384	63.87 ± 1.44
NLC 9	2:1	0.2	Cremophor^®^ RH40	321.56 ± 87.44	0.17 ± 0.18	−33.950 ± 0.354	34.31 ± 1.26
NLC 10	1.5:1	0.2	Solutol^®^HS15	162.65 ± 10.67	0.41 ± 0.02	−36.750 ± 0.495	47.86 ± 7.96
NLC 11	2:1	0.6	Cremophor^®^ RH40	193.05 ± 23.82	0.35 ± 0.02	−34.250 ± 2.051	45.07 ± 2.81
NLC 12	1:1	0.4	Solutol^®^HS15	191.30 ± 37.61	0.48 ± 0.08	−36.300 ± 4.384	70.64 ± 0.00
NLC 13	1:1	0.2	Solutol^®^HS15	114.30 ± 6.64	0.42 ± 0.08	−32.350 ± 1.202	59.31 ± 0.05
NLC 14	2:1	0.6	PL^®^-F127	100.85 ± 0.49	0.20 ± 0.11	−28.850 ± 0.071	57.56 ± 0.23
NLC 15	1.5:1	0.2	Cremophor^®^ RH40	230.23 ± 179.72	0.25 ± 0.04	−32.350 ± 1.626	49.16 ± 12.15
NLC 16	1:1	0.6	Solutol^®^HS15	230.95 ± 28.07	0.44 ± 0.03	−34.150 ± 1.626	48.41 ± 8.84
NLC 17	2:1	0.6	Solutol^®^HS15	169.45 ± 52.11	0.36 ± 0.08	−31.350 ± 0.636	54.05 ± 0.44
NLC 18	2:1	0.6	Cremophor^®^ RH40	212.65 ± 5.30	0.32 ± 0.05	−35.000 ± 0.283	36.97 ± 6.38
NLC 19	2:1	0.2	PL^®^-F127	125.20 ± 32.38	0.33 ± 0.07	−20.200 ± 1.273	49.15 ± 4.17

All formulations contain EPL 25 mg/20 mL. Data are represented as (mean ± SD).

### 2.5. *In vitro* characterization of the optimized EPL-loaded NLC system

The optimized EPL-loaded NLCs system was prepared and evaluated for the PS, PDI, ZP, and EE%. The residual difference between the predicted and the measured responses was measured to ensure the validity of the design (Sayed et al., 2018).

### 2.6. *In-vitro* drug release study

The cumulative % EPL released from the optimized EPL-loaded NLCs system was performed using dialysis bag technique (Üner et al., 2014). Briefly 2 mL of the NLCs system (equivalent to 2.5 mg EPL) were placed in a dialysis bag and the ends of dialysis bag were sealed with standard closures, and then immersed in 100 mL phosphate buffer solution (PBS pH 7.4) in a tightly closed flask kept in thermostatically controlled water bath shaker (Gesellschatt Laboratories, Berlin, Germany) at 37 ± 0.5 °C and shaken at 100 rpm. At different time intervals (0.25 h, 0.5 h, 1 h, 1.5 h, 2 h, 4 h, 6 h, 8 h, and 24 h), 3 mL sample were withdrawn from the release medium and replaced by equal volume of fresh medium. For comparison, EPL aqueous suspension (2.5 mg/2 mL) was prepared and EPL release was determined using the same procedure mentioned above and compared to that of the EPL-loaded NLCs optimized system. Samples were analyzed using UV-spectrophotometry at *λ*_max_ 245 nm using UV/VIS spectrophotometer (Shimadzu, UV-1601 PC, Kyoto, Japan).

### 2.7. Morphological evaluation of the optimized EPL-loaded NLCs system

Morphological parameters of the optimized EPL-loaded NLCs system were observed using transmission electron microscopy (TEM) at 70 kV (JEOL JEM1230, Tokyo, Japan). The optimized EPL-loaded NLCs system was first diluted with 10-fold its volume distilled water, then added to copper grid coated with collodion film after being stained by 2% (w/v) phosphotungestic acid solution (negative stain) and dried at room temperature, then observed under the TEM.

### 2.8. Fourier infrared spectroscopy and X-ray diffraction

The FTIR spectroscopy study was employed using a Bruker FTIR spectrophotometer (Model 22; Bruker, Coventry, UK) to detect the chances of any chemical interactions between EPL and other ingredients used in the preparation of the optimized NLCs system. The IR spectra of plain EPL, physical mixture (composed of EPL, GMS and Pluronic^®^ F127 in equal amounts) and the freeze-dried optimized EPL-loaded NLCs system were recorded. Samples were mixed with KBr and compressed into disk, then scanned in the range 4000–400 cm^−1^ at ambient temperature. For freeze-drying, EPL-loaded NLCs system was first frozen at −22 °C for 24 h and then placed in the freeze-dryer (Novalyphe-NL 500, Halprook, NY) for 24 h. The freeze-dryer was operated under vacuum at pressure of 7 × 10^−2^ mBAR. The condenser temperature was adjusted at − 45 °C.

X-ray powder diffraction (X-RPD) experiments were carried out using X-ray diffractometer (XGEN-4000, Scintag Corp., Sunnyvale, CA), with Cu Ka radiation at 40 mA current and 45 kV Voltage. Diffraction patterns for plain EPL, physical mixture (composed of EPL, GMS, and Pluronic^®^ F127 in equal amounts) and the freeze-dried optimized EPL-loaded NLCs system were recorded as the X-ray intensity as a function of 2*θ* angle covering from 2.0° to 50.0°. The scanning rate was 6°/minute. The X-ray diffraction patterns were displayed using Diffrac AT software.

### 2.9. *Ex vivo* permeation study

#### 2.9.1. Animals

Male albino rabbits (2.5–3 kg) were housed in a controlled temperature and humidity room (25 °C, 55% air humidity with free access to water ad libitum). Experiments were approved by the Research Ethics Committee (REC; PT 2682) at Faculty of Pharmacy, Cairo University (Cairo, Egypt). One day before the experiment, rabbits were fasted overnight with free access to water. Rabbits received an intramuscular injection of ketamine (35 mg/kg) as an anesthetizing agent and 5 mg/kg xylazine as a relaxing agent, then sacrificed and the small intestine was removed by a midline abdominal incision. The small intestine cleaned carefully using a syringe filled with warm (37 °C) saline (pH = 7.4), then the intestinal segments (∼7.8 cm^2^) were separated.

#### 2.9.2. Study design

Accurately, 4 mL of EPL-loaded optimized NLCs system (equivalent to 5 mg of drug) were filled in an intestinal segment (of area 7.8 cm^2^) via micropipette, then the two sides of the intestine were tangled with a thread. Each intestinal sac was placed in a bottle containing 500 mL of normal saline (pH = 7.4) maintained at 37 ± 0.5 °C. The bottle was placed in a thermostatically controlled shaking water bath (GFL, Gesellschatt Laboratories, Berlin, Germany) maintained at 37 ± 0.5 °C and 100 rpm. Samples (3 mL) were withdrawn at specific time intervals (0.5 h, 1 h, 2 h, 4 h, 6 h, 8 h, and 24 h) and replaced by equal volume of fresh medium. In order to determine the amount of EPL permeated through the intestine, samples were diluted with equal amount of acetonitrile (HPLC grade) followed by sonication for 10 min in order to detect the total drug permeated (as free and vesicles-encapsulated drug). Samples were analyzed according to a previously validated HPLC method (Rane et al., 2009). For comparison, EPL permeation from EPL aqueous suspension containing EPL 1.25 mg/mL was examined using the same procedure mentioned above.

Chromatographic separation was achieved on RP C_18_ column (250 mm × 4.6 mm, 5 μm). The system consisted of an Agilent 1260 infinity series connected to a DAD detector with a quaternary pump and an injector with a 20 μl loop. Manual injections were carried out using a 100 μl Hamilton syringe. The mobile phase consisted of a mixture acetonitrile and ammonium acetate buffer 50 mM adjusted at pH 7.0 (at ratio 55:45 v/v). The determination was performed using ultraviolet detector (Shimadzu, Tokyo, Japan) at 240 nm and injection volume was 5 μL.

## 3. Results and discussion

### 3.1. Selection of EPL-loaded nanostructured lipid matrix

EPL-loaded NLCs were successfully prepared using the emulsification solvent evaporation method. All the prepared systems were homogenous and showed no signs of precipitation or phase separation. GMS was chosen as solid lipid to prepare EPL-loaded NLCs, owing to its inherent self-emulsifying property and low cytotoxicity. Solid lipids of long hydrocarbon chain (more than C_12_) are characterized by low HLB values and high solubilizing power so they are preferred as lipid excipients (Patil-Gadhe & Pokharkar, 2014). Miglyol^®^ 812N was selected as the liquid lipid, being a medium chain triglyceride with a unique class of saturated lipids, in addition to its ability to act as emulsifier and suspender. It is well known that medium chain triglycerides excipients composed of C_6–12_ fatty acids, such as Miglyol^®^ 812N are easily absorbed from the intestine (Furuse et al., 1992). Regarding the surfactants, Pluronic^®^ F127, Solutol^®^HS15, and Cremophor^®^ RH40 were evaluated as nonionic surfactants in the preparation of EPL-loaded NLCs, due to their low toxicity and high hydrophilicity and compatibility. Further, the used solvent mixture (ethanol:acetone mixture) was selected based on its ability to solubilize drug, solid lipid, and liquid lipid results in their thorough and uniform drug–lipid association (Hu et al., 2002, 2005).

### 3.2. Analysis of the factorial design

For analysis of model parameters, the model having high values of adjusted *R*^2^ and predicted *R*^2^, within 0.2 numerical value difference between each of other was considered to ensure the validity of the analyzed model. The model should have nonsignificant lack of fit with adequate precision more than 4 (Huang et al., 2004). Table 3 shows the regression analysis for the studied factors and the best model selected through the design expert ^®^software (design expert software version 7).

**Table 3.** Significance of different model terms appearing in the final reduced model for each response, along with the selected model and its evaluation.

Term	*p* Value		
	PS	PDI	EE
A	.001	˂.0001	.0001
B	.0479	–	–
C	.0001	–	˂.0001
AB	˂.0001	.0949	0.0007
AC	˂.0001	.0003	–
BC	.003	˂.0001	–
B^2^	–	–	.0002
Lack of fit	.5240	.4571	.8118
Model	2FI	2FI	Quadratic
*R* ^2^	.9639	.9417	.9162
Adjusted *R*^2^	.9277	.9125	.8840
Prediction *R*^2^	.8274	.8595	.8254
Adequate precision	18.635	22.088	18.648
PRESS	1319.01	0.034	360.79

A: liquid lipid to solid lipid ratio, B: concentration of surfactant, C: type of surfactant, and *R*^2^ is a regression coefficient.

### 3.3. Effect of variables on the properties of the prepared EPL-loaded NLCs

#### 3.3.1. Particle size of EPL-loaded NLCs

Table 2 shows the results PS of EPL-loaded NLCs characterization. The average PS of the prepared EPL-loaded NLCs ranged from 100.85 ± 0.49 to 346.60 ± 16.82 nm. The 2FI model was the most suitable one fitting (*p* value˂.0001) with non-significant lack of fit (*p* value = .5240, Table 3) and adequate precision of 18.635 indicating the model ability to navigate the design space (Elbary et al., 2011).

**Figure 1.**
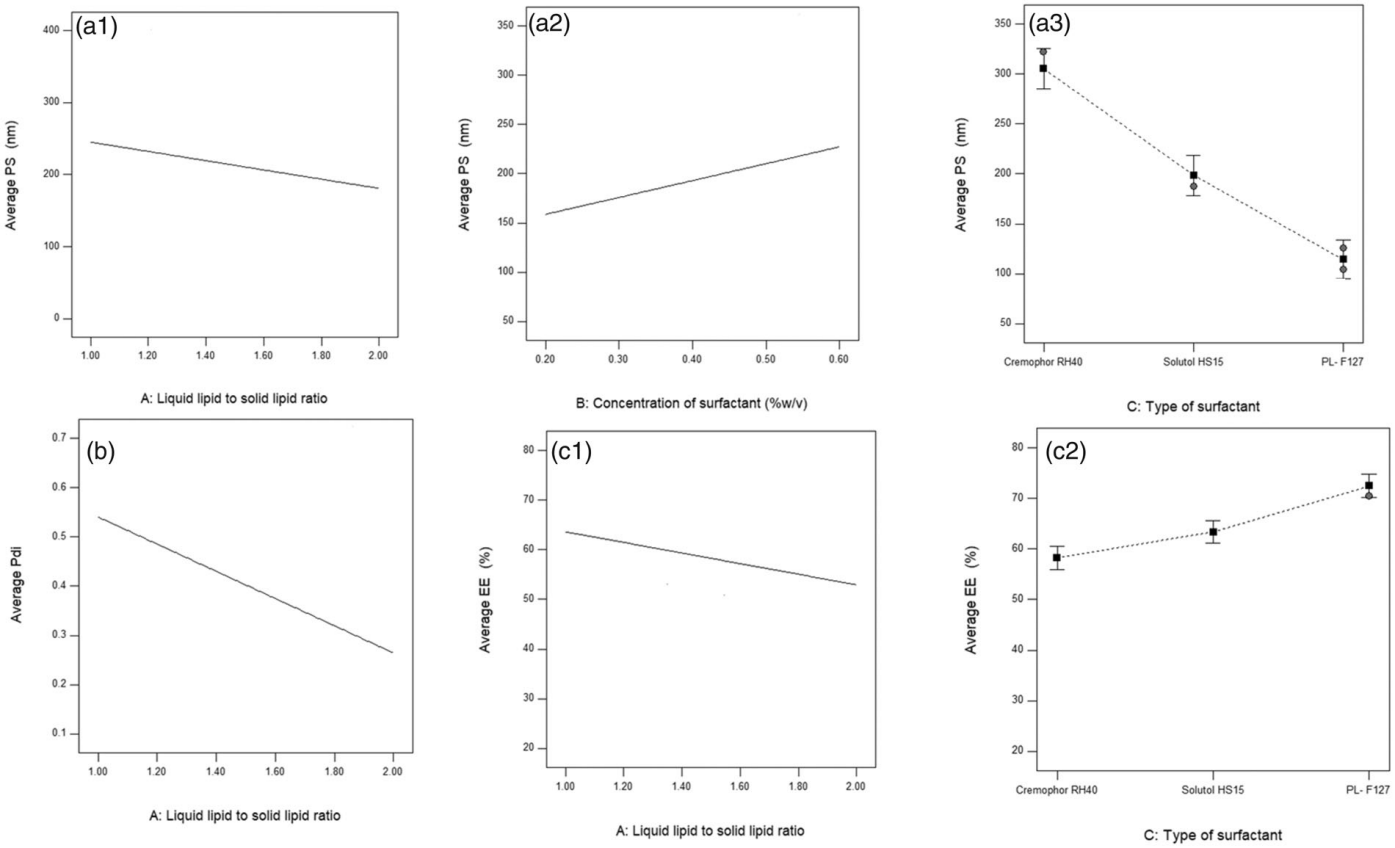
(a) Effect of liquid lipid to solid lipid ratio a1, surfactant concentration a2, and surfactant type a3 on PS, (b) effect of liquid lipid to solid lipid ratio on PDI, and (c) effect of liquid lipid to solid lipid ratio c1 and surfactant type c2 on EE% of the prepared EPL-loaded NLCs systems.

All the studied factors had significant effect on the PS (Table 3). ANOVA results showed that increasing the liquid lipid to solid lipid ratio led to a significant decrease in the PS of the prepared EPL-loaded NLCs (*p* = 0.001), Figure 1(a_1_). This is could be attributed to the nature of the used liquid oil (Miglyol^®^812N), which is considered as an emulsifier and suspender. The low viscosity of Miglyol^®^812N allows the rapid diffusion of the organic phase through the aqueous phase during the solvent evaporation, where the surfactant molecules move faster and, therefore, produce smaller droplets (Wang et al., 2014). These results are in accordance with previously published results by Pezeshki et al. (Hasan et al., 2017), in their study on the preparation of betacarotene-loaded nanoemulsion. The authors reported that the smallest PS was observed upon using Miglyol^®^ 812N compared to other oils (corn oil and octyl octanoate). The mechanism of PS reduction by Miglyol^®^ 812N could be attributed to the reduction of the interfacial tension owing to its surfactant property (Sanad et al., 2010).

ANOVA results also showed that increasing the concentration of the surfactant is associated with a significant increase in the PS (*p* = .0479), Figure 1(a_2_). The surfactant concentration affects the PS in a paradoxical manner, where increasing the surfactant concentration leads to faster adsorption on particle surface making a mechanical barrier against crystallization and, thus, a smaller final PS. However, excess surfactant would increase the PS as well (Hasani et al., 2015), where above the critical micelle concentration, the surfactant molecules are oriented in a micelle form rather than to be adsorbed on other surfaces. Hence, the surfactant adsorption on particle surface is reduced. As a result, particle agglomeration is increased leading to larger PS. Also above the critical micelle concentration, the micelle solubilization may solubilize some of the drug particles and thereby enhances the particle growth by the Ostwald ripening process (Deng et al., 2010). A similar finding was reported by Shamma et al. (Aburahma et al., 2014), in their study on the fabrication of NLCs for follicular delivery of spironolactone. The authors reported a concentration-dependent increase in PS with the increase in surfactant concentration (Tween 80) and related this finding to the hydrophobic interaction between the nonpolar alkyl chains of the surfactant and the solid lipid molecules resulting in larger PS. Similar results were also obtained by Kumbhar & Pokharkar (2013), in their study on the preparation of bicalutamide-loaded NLCs, where an increase in PS was reported upon increasing the Pluronic^®^ F127 concentration.

**Figure 2.**
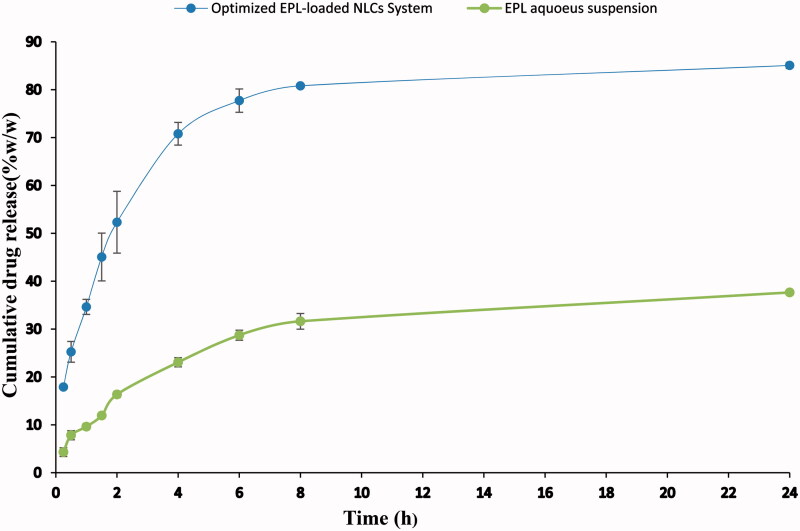
Cumulative release profile of EPL from the optimized EPL-NLCs system compared to EPL aqueous suspension.

Concerning the surfactant type, ANOVA results showed that the type of surfactant had a significant effect on the PS (*p* = .0001). The PS of EPL-loaded NLCs prepared using Pluronic^®^ F127 were significantly smaller compared to NLCs prepared using Solutol^®^ HS15 and Cremophor^®^ RH40. as illustrated in Figure 1(a_3_). Cremophor^®^RH40 is a bulky surfactant possessing polyethylene glycols and glycerol ethoxylates of long-chain ricinoleic acid. This could have imparted poor stabilizing efficiency and affect its ability to produce small PS (Shete & Patravale, 2013). On the other hand, Pluronic^®^F127 imparted better surface coverage and enabled smaller PS. Pluronic^®^F127 is a stabilizer with a big hydrophilic head group, which provides a better steric hindrance and thus reduces the PS. The results are in agreement with Weerapol et al. (2014) who reported that the molecular structure of the surfactant has a significant effect on the final droplet size .

**Figure 3.**
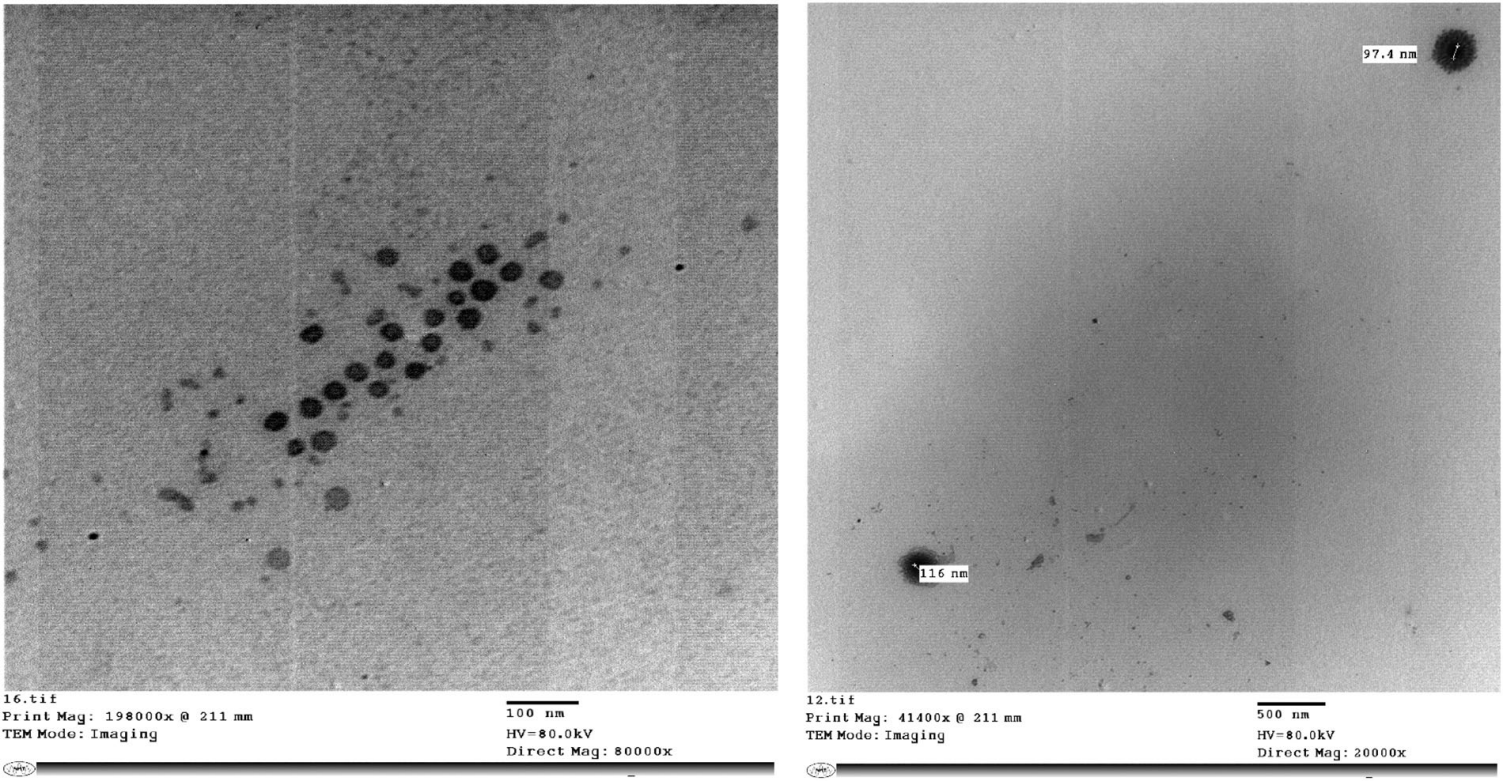
TEM images of the optimized EPL-loaded NLCs system.

#### 3.3.2. Polydispersity index of EPL-loaded NLCs

Table 2 shows the PDI values of the prepared EPL-loaded NLCs. PDI values ranged from 0.17 ± 0.18 to 0.62 ± 0.03 indicating that most of the measured NLCs systems have an acceptable homogenous particle distribution. The 2FI model was the most suitable model fitting (*p* value ˂ .0001) with non-significant lack of fit (*p* value= .4571) and adequate precision of 22.008 indicating an adequate signal (Table 3).

ANOVA results showed that only the liquid lipid to solid lipid ratio had a significant negative effect on the PDI (*p* value˂.0001), Increasing the ratio was associated with significant reduction in the PDI value, Figure 1(b). Generally, the addition of liquid lipid (Miglyol^®^812N) favored the formation of homogeneous particles (Aburahma et al., 2014). The higher content Miglyol^®^812N reduces the viscosity inside NLCs, and reduces the surface tension resulting in the formation of smaller and smoother surface particles with low PDI (Jenning et al., 2000; Uprit et al., 2013). Similar results were obtained by several researchers in the literature (Hu et al., 2005; Agrawal et al., 2010), where the PDI of the prepared NLCs was decreased by increasing the liquid lipid (oleic acid) content.

#### 3.3.3. Zeta potential of EPL-loaded NLCs

The ZP provides information about the magnitude of the charge on the surface of the particles in an aqueous dispersion and allows predicting the long-term physical stability of the formulations (Kovačević et al., 2014). Results of ZP of the prepared EPL-loaded NLCs are presented in Table 2. Results show that the prepared EPL-loaded NLCs were all negatively charged having ZP in the range of −20.200 ± 1.273 to −36.750 ± 0.495 mV. Generally, the lipid nanocarriers are negatively charged because of their high content of fatty acids and triglycerides (Schwarz & Mehnert, 1999; Shah et al., 2014). Patil et al. (2016). reported that GMS used in the formulation of carvedilol-loaded NLCs resulted in particles with negative ZP. In addition, the used liquid lipid, Miglyol^®^812N, carry negative charge at the carboxylic groups imparting a negative surface charge (Harisa & Badran, 2015). These results are in agreement with those obtained in a previous study by Hou et al. (2003).

The ZP has a negative predicted *R*^2^ value indicates that the overall mean is a better predictor of this response and it was not significantly affected by the studied factors.

#### 3.3.4. Entrapment efficiency of EPL-loaded NLCs

The EE% of EPL in prepared EPL-loaded NLCs are presented in Table 2. The EE% ranged from 34.31 ± 1.26 to 70.64 ± 0.00% w/w. The Quadratic model showed the highest *R*^2^, thus, it was selected for analyzing the effect of variables on the EE% (*p* value˂.0001) with non-significant lack of fit (*p* value = .8118) and adequate precision of 18.648 (Table 3).

ANOVA results showed that the liquid lipid concentration had a significant negative impact on EPL EE%. Increasing the liquid lipid:solid lipid ratio is associated with a significant reduction in the EE% of EPL in the prepared NLCs (*p* = .0001), Figure 1(c_1_). The ratio of components (solid lipid and liquid lipid) has been reported to significantly impact the final structure of the lipid particles and hence the loading of the molecule (Uprit et al., 2013). At high ratios of oil, the drug molecules might be expulsed from the inner core of the solid matrix toward the surface of the particle, because the limits of solubility could be exceeded. The same can be said about the use of high ratios of stabilizing agents, which may generate solubilized systems rather than particulate systems where the molecules of the lipid components could not form solid structures (Bunjes et al., 1996). Another explanation is that, during NLCs fabrication, all or part of the lipid matrix re-transformed to a less or unstable α-polymorphic form or to the metastable β’-polymorphic form then to the most stable β-form (Westesen et al., 1993). During this transformation, there is a subsequent reduction in the number of defects in the lipid matrix, as in the case of the configuration of β’/β forms leading to diminishing the drug encapsulation. The rate of this conversion is higher in short- and medium chain ones (like Miglyol^®^812N) compared to for long-chain triglycerides (Souto & Müller, 2010).

ANOVA results also showed that the type of surfactant had a significant impact on the EE% of EPL in the prepared EPL-loaded NLCs (*p* < .0001), Figure 1(c_2_). The highest EE% was observed when using PL^®^F127 as surfactant followed by Solutol^®^HS15 and the lowest one observed with Cremophor^®^RH40.

An obvious variation in emulsification power was evident upon using the previously mentioned surfactants despite the fact that all of them have HLB values greater than 10. This is because various factors rather than HLB value had also a great impact on the magnitude of EE% like the length and structure of hydrocarbon chain of surfactants. Cremophor^®^RH40 is composed mainly of castor oil, a triglyceride in which each of its three hydroxyl groups esterifies with a long-chain unsaturated fatty acid (hydroxylated 12-hydroxy, 9-octadecenoic acid) known as ricinoleic acid. Glycerol ethoxylates and polyethylene glycols derivatives of long-chain ricinoleic acid were found to impart poor stabilizing efficiency confirmed by the lower EE% at higher concentration especially in the presence of low lipid concentration (Abd El-Halim et al., 2020). On contrary, Pluronic^®^F127 being block copolymer is composed of polyoxypropylene oxide as a hydrophobic segment and polyoxyethylene oxide as a hydrophilic segment (Dai et al., 2008; Ghosh et al., 2011). This huge polymeric grid of polyoxyethylene oxide (hydrophilic segment) orients in external phase, while polyoxypropylene oxide (hydrophobic segment) settles at interface and leading to more steric stabilization of NLCs permitting larger surface area for drug housing (Chen et al., 2014). Another explanation, Pluronic^®^ F127 has a higher nominal molecular weight (12,500) g/mol (Bohorquez et al., 1999) than that of Cremophor^®^RH40 (2500 g/mol) (Zeng et al., 2017), therefore, Pluronic^®^ F127 can form rigid dispersion with low tendency of polymorphic transition. It is known that polymorphic transition reduces drug loading capacity in NLCs due to increased ordering and packing density of the lipid crystals, thus, leading to expulsion of drug from the crystal matrix and consequently low entrapment (Bohorquez et al., 1999). Unlike Pluronic^®^ F127, Cremophor^®^ RH40 is semisolid at room temperature, so the formed surfactant layer is relatively flexible, and, thus, likely induced rapid polymorphic transition.

## 4. Optimization and validation of EPL-loaded NLCs

The optimization process depends on the collection all the responses into one variable in order to elucidate the desired levels for each of the studied factors (Pandya et al., 2011). The optimum NLCs system with smallest PS, highest EE%, and PDI less than 0.5 with desirability value equal 0.905 was suggested through Design expert^®^ software. This optimized EPL-loaded NLCs system is composed of liquid to solid lipid ratio equal 2:1 in presence of 0.43%w/v PL-F127 as a surfactant. Accordingly, this NLCs system was prepared and evaluated for the PS, PDI, ZP, and EE%. Table 4 shows the predicted values for all responses of the optimum formula, compared to actual values of the prepared optimized one and the calculated residual value. Results show that the actual values close to the predicted values indicating the success of the design.

**Table 4.** Predicted values of the measured responses for the optimized formula compared to the actual values along with the prediction intervals and the residual value.

		Two-sided prediction interval			
	Predicted values	Low	High	Actual values	Residual value
PS (nm)	111.16	64.79	157.54	134	22.83
PDI	0.25	0.17	0.34	0.31	0.05
EE (%w/w)	67.41	57.18	77.66	76	8.58

The Optimized system composed of liquid lipid to solid lipid ratio 2:1 in presence of 0.43%w/v Pluronic^®^F127.

### 4.1. *In-vitro* comparative drug release study

Figure 2 shows the release profile of EPL from the optimized EPL-loaded NLCs system compared to that of EPL drug suspension (containing equivalent drug dose as the optimized system). As shown in Figure 2, the optimized NLCs system succeeded to control the release of EPL with about 52.31% EPL released after 2 h compared to 16.34% EPL released from the aqueous suspension at the same time. By the end of the release time (24 h), 85.064% and 37.65% EPL were released from the optimized NLCs system and the aqueous suspension, respectively. This difference in the release pattern is due to the fact that most NLCs exhibit a biphasic release pattern (Abd El-Halim et al., 2020; Gordillo-Galeano & Mora-Huertas, 2018; Yu et al., 2018;  Joshi and Patravale 2006); starts with a spurt stage due to the rapid release of the surface drug in the outside shell, while the second prolonged phase (up to 24 h) is due to the presence of the encapsulated drug within the lipid matrices. Moreover, the nature of the stabilizing agent (Pluronic^®^F127) used and its arrangement (Abdelbary & Haider, 2013; Dan, 2014; Alvarez-Trabado et al., 2017), greatly affect the drug release pattern by providing large surface area for drug accommodation, consequently hinders the drug expulsion from the NLCs and imparts great stability (Ghosh et al., 2011).

### 4.2. Transmission electron microscopy (TEM)

TEM images of the optimized EPL-loaded NLCs, presented in Figure 3, revealed that the particles were non-aggregated with smooth spherical shape and narrow size distribution. These results are in accordance to previously obtained studies showing that NLCs are spherical in shape (Saupe et al., 2006; Araújo et al., 2010; Alam et al., 2016). Also, it can be noticed that some of the obtained NLCs particles had an empty core and a drug-enriched shell demonstrated by the dark shade around the particles confirming the ability of the used surfactant to solubilize the drug and concentrate it on the surface of the nanoparticles. Results of the PS obtained from the images are in close agreement with those obtained by the dynamic light scattering.

### 4.3. Fourier infrared spectroscopy and X-ray diffraction

**Figure 4.**
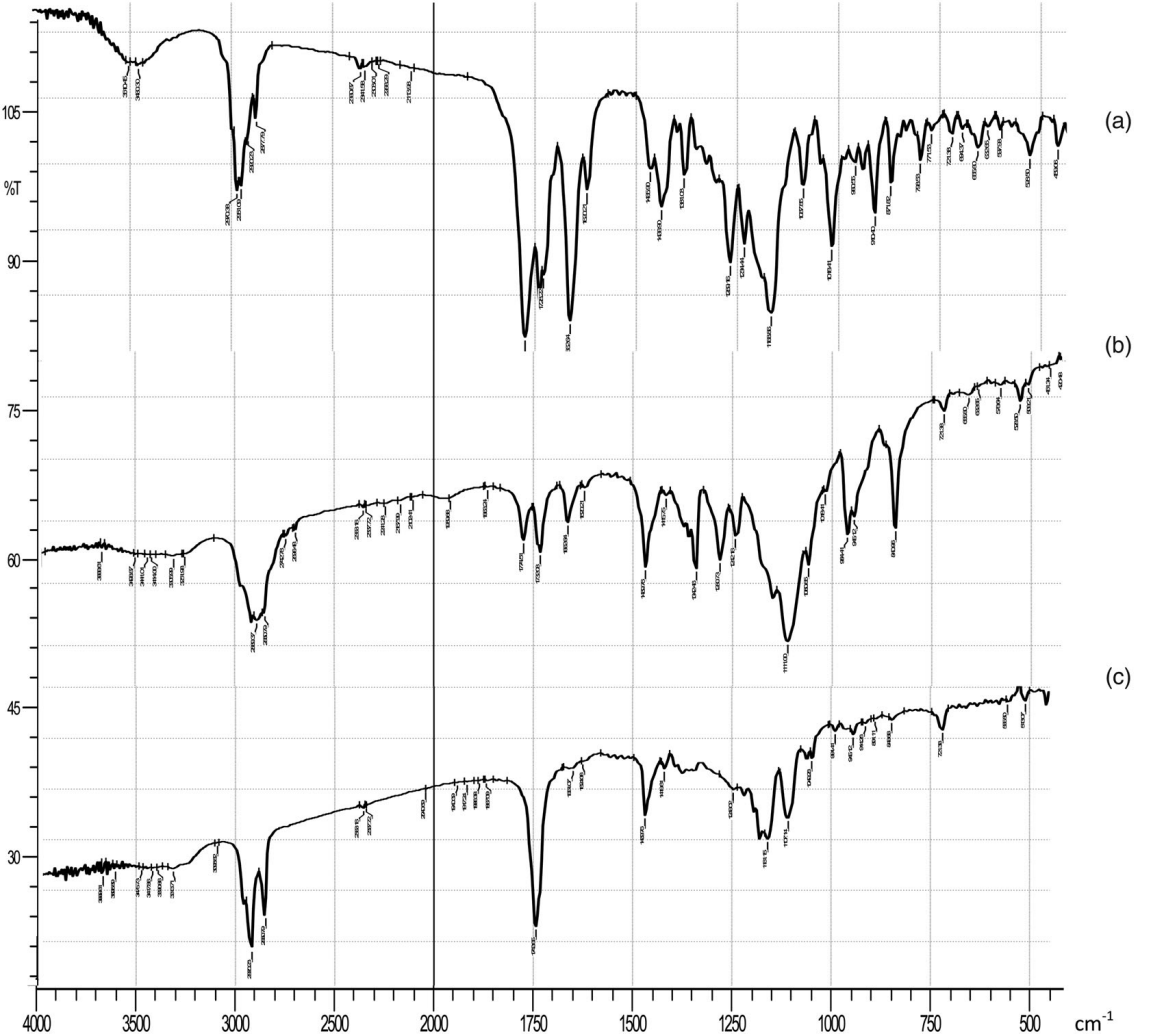
IR spectrum of (a) plain EPL, (b) physical mixture, and (c) freeze-dried optimized EPL loaded-NLCs system.

Figure 4 presents the IR spectra of the tested samples. The IR spectrum of pure EPL shows the main characteristic functional groups at 2988.65 cm^−1^ (C–H stretching), 1778.08 cm^−1^ (anhydride O–C=O stretching), 1726.22 cm^−1^ (C=O ester stretching), and (1657.64 cm^−1^) C=O stretching. IR spectra of the physical mixture and the freeze dried EPL-loaded NLCs system showed no chemical changes, where all drug functional groups were retained, which confirmed the compatibility of EPL with other formulation additives.

**Figure 5.**
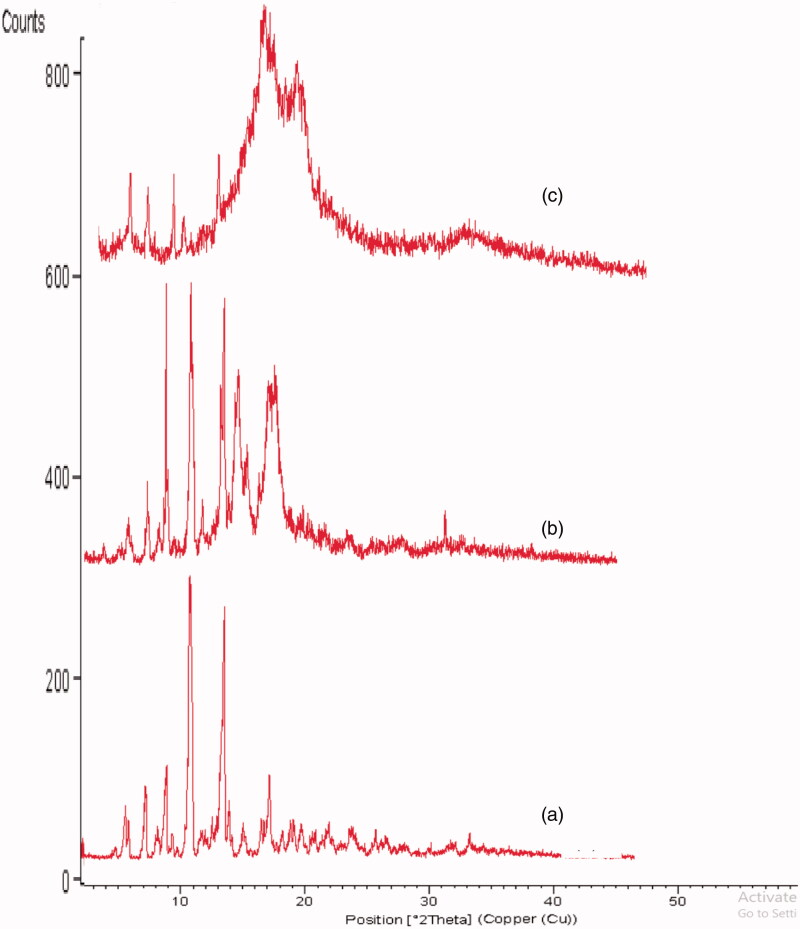
X-ray diffractogram of (a) plain EPL, (b) physical mixture, and (c) freeze-dried optimized EPL loaded-NLCs system.

Figure 5 presents the diffraction patterns of the examined samples. The diffraction pattern of pure EPL exhibited high-intensity crystallinity peaks at 10.19, 12.24, 14.55, 18.12, and 22.61° 2*θ*. In the diffractogram of the physical mixture, the characteristic peaks of GMS were present at 11.9, 14.58, and 18.00° 2*θ*, confirming its crystallinity. The crystalline peaks of EPL in physical mixture at 10.19, 14.55, and 22.61° 2*θ* were evident but with lower intensities, confirming the presence of EPL in crystalline form. On the contrary, the diffractogram of optimized EPL-loaded NLCs system showed absence of EPL constructive peaks, signifying that the drug lost its crystallinity where it was transformed to an amorphous state or dispersed within the nanocarriers. On the other hand, the two characteristic peaks of GMS (14.58, 18.00° 2*θ*) were still present in the diffractogram of optimized NLCs system confirming that the carrier crystallized into its stable polymorphic form.

### 4.4. *Ex-vivo* permeation study

**Figure 6.**
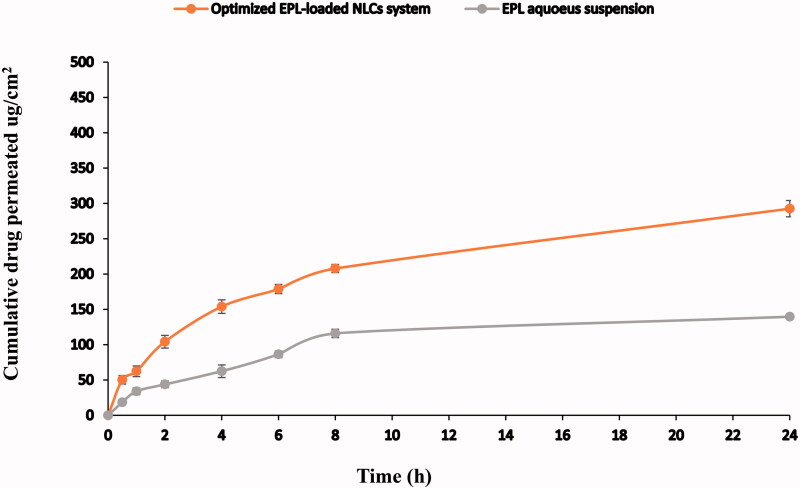
*Ex vivo* permeation profiles of EPL from optimized EPL-NLCs system (containing 5 mg EPL) through intestinal membrane in saline solution compared to EPL aqueous suspension.

The HPLC method showed a linear response in the range of 0.2–2 μg/ml (*R*^2^ = 0.9915). Figure 6 shows the *ex-vivo* permeation profiles of EPL through the rabbit intestine in 0.9% saline (pH = 7.4) from the optimized EPL-loaded NLCs (containing 5 mg EPL) compared to that of EPL aqueous suspension (containing an equivalent dose). As shown in Figure 6, great difference in the permeation profiles of optimized EPL-loaded NLCs and dug aqueous suspension through the intestine was observed, where the cumulative amount of drug permeated from the optimized EPL-loaded NLCs was two-fold higher that that from EPL aqueous suspension after 24 h. The optimized EPL-loaded NLCs showed significantly higher cumulative amount of EPL permeated after 24 h (Q_24h_ optimized NLCs system∼292.56 ± 11.61 μg/cm^2^) compared to (Q_24h_ suspension∼139.35 ± 0.00 μg/cm^2^) (*p* < .05). The low transfer through the intestine was observed with drug suspension due to the poor EPL aqueous solubility (BCS class II) (Khames, 2019).

The transport of drug from optimized-loaded NLCs system through intestinal membrane could be achieved as free molecules or as the intact vesicles encapsulating the drug that was acetonitrile diluted samples were used for the analysis (acetonitrile is capable to disrupt the vesicle wall releasing the encapsulated drug) (Ibrahim et al., 2020). Several studies reported the uptake of intact vesicles by the intestinal epithelial cells (M-cells) in the Payer's patch (Poonia et al., 2016; Aburahma, 2014) and the intestinal enterocytes (Niu et al., 2014), confirming that the main transport mechanism is supposed to be through vesicular uptake as most of the drug is encapsulated into the vesicles, where the free nanosized molecules are transported and absorbed via paracellular or transcellular pathways through the intestinal membrane. These results indicate that the optimized NLCs system was capable to incorporate high amounts of drug and, hence, achieve high drug permeation in the *ex-vivo* study.

## 5. Conclusion

In this study, EPL-loaded NLCs were successfully prepared by emulsification-solvent evaporation method using d-optimal design. The optimized system exhibited a small particle size (134 ± 15.60 nm) with narrow size homogenous distribution (PDI = 0.313 ± 0.09), and high entrapment efficiency (76 ± 6.56%). Morphological examination by TEM confirmed a small smooth spherical nano-carrier structure. The *ex-vivo* permeation study confirmed the ability of the optimized system to cross the intestinal barrier, with two-fold higher drug permeated after 24 h compared to the drug aqueous suspension. These promising results pave the way for the potential use of the prepared EPL-loaded NLCs as s successful drug delivery system for oral treatment of CSCR.
